# Livestock trade networks for guiding animal health surveillance

**DOI:** 10.1186/s12917-015-0354-4

**Published:** 2015-04-01

**Authors:** Jo L Hardstaff, Barbara Häsler, Jonathan R Rushton

**Affiliations:** University of Liverpool- Institute of Infection and Global Health, The Farr Institute@HeRC, 2nd Floor - Block F, Waterhouse building, Liverpool, L69 3GL UK; Leverhulme Centre for Integrative Research on Agriculture and Health, Royal Veterinary College, Hawkshead Lane, North Mymms, Hatfield, Hertfordshire, AL9 7TA UK; Department of Production and Population Health, Royal Veterinary College, Hawkshead Lane, North Mymms, Hatfield, Hertfordshire, AL9 7TA UK

**Keywords:** Livestock, European Union, Transport, Surveillance

## Abstract

**Background:**

Trade in live animals can contribute to the introduction of exotic diseases, the maintenance and spread endemic diseases. Annually millions of animals are moved across Europe for the purposes of breeding, fattening and slaughter. Data on the number of animals moved were obtained from the Directorate General Sanco (DG Sanco) for 2011. These were converted to livestock units to enable direct comparison across species and their movements were mapped, used to calculate the indegrees and outdegrees of 27 European countries and the density and transitivity of movements within Europe. This provided the opportunity to discuss surveillance of European livestock movement taking into account stopping points en-route.

**Results:**

High density and transitivity of movement for registered equines, breeding and fattening cattle, breeding poultry and pigs for breeding, fattening and slaughter indicates that hazards have the potential to spread quickly within these populations. This is of concern to highly connected countries particularly those where imported animals constitute a large proportion of their national livestock populations, and have a high indegree. The transport of poultry (older than 72 hours) and unweaned animals would require more rest breaks than the movement of weaned animals, which may provide more opportunities for disease transmission. Transitivity is greatest for animals transported for breeding purposes with cattle, pigs and poultry having values of over 50%.

**Conclusions:**

This paper demonstrated that some species (pigs and poultry) are traded much more frequently and at a larger scale than species such as goats. Some countries are more vulnerable than others due to importing animals from many countries, having imported animals requiring rest-breaks and importing large proportions of their national herd or flock. Such knowledge about the vulnerability of different livestock systems related to trade movements can be used to inform the design of animal health surveillance systems to facilitate the trade in animals between European member states.

**Electronic supplementary material:**

The online version of this article (doi:10.1186/s12917-015-0354-4) contains supplementary material, which is available to authorized users.

## Background

Animal trade is an effective way of introducing, maintaining and spreading animal diseases, as observed with the spread of different strains of foot and mouth disease (FMD) in Africa, the Middle-East and Asia [1] and the spread of bovine spongiform encephalopathy (BSE), for example into Oman and Canada through the importation of infected cattle [2,3]. Within a year, millions of live animals of many different species are transported between countries within Europe for breeding, fattening, sports, companionship, conservation and slaughter. This creates opportunities for communicable diseases to be spread across the European Union (EU), which is the focus of this study, even though animals must be in a fit state to be transported i.e. healthy animals without clinical signs of illness [4]. However, animals with sub-clinical infections may go unnoticed, providing an opportunity to transport disease to different regions. Live animal trade complicates tracing the origin of any disease outbreak that may occur due to an infected animal being displaced. For this reason, the EU has established a Trade Control and Expert System (TRACES) to monitor imports, exports and trade in animals and animal products across the EU and to ensure traceability within the food chain [5], in addition to livestock movements recorded by the Food and Agricultural Organisation of the United Nations (FAO). TRACES records the number of animals and consignments entering and leaving EU countries. Despite the availability of this comprehensive database, animal health surveillance systems are rarely based on international live animal movements. To understand better livestock trade within Europe with a view to inform disease surveillance we analysed trade networks across the EU for all major livestock species and purposes of movements.

Animal health surveillance includes the systematic, continuous or repeated, measurement, collection, collation, analysis, interpretation and timely dissemination of animal health and welfare related data from defined populations, essential for describing health hazard occurrence and to contribute to the planning, implementation and evaluation of risk mitigation measures [6]. Recent outbreaks and spread of exotic or emerging diseases such as avian influenza (AI), Schmallenberg virus (SBV) and bluetongue virus (BTV) in previously unaffected territories of the EU have emphasised the need for well-developed and adequately resourced health systems, including surveillance, to ensure early detection and rapid containment, the complexities of which are highlighted by Braks et al. (2011) [7]. At the same time investment is being constrained due to significant financial budget reductions in many European countries. Livestock disease is important economically with regards to a loss of productivity, its potential impact on human and animal health, and the mitigation activities implemented when disease occurs (for example trade or movement bans, testing and culling). For example, the economic cost of BSE in the UK accrued from the value loss in infected carcasses, disposal costs, and, most importantly, the sharp drop in domestic beef demand due to consumer scares (sales of beef products declined by 40% once the possible link between BSE and new variant Creutzfeldt-Jakob disease (CJD) was announced, but the costs were partly offset by an increase in consumption of substitute meat), and a complete loss in export markets [8]. Further costs accrued from operating various public schemes, establishment and enforcement of new legislation and the adjustment of the industry to the new structure and markets [8]. Livestock disease can be spread directly for example the introduction of FMD from Irish calves imported to the Netherlands that were also held responsible for the infection of a farm near to the port of introduction to mainland Europe [9]. It can be spread by infected equipment, crates or transporter vehicles which can be contaminated by microbes. For example *Escherichia coli* (*E. coli*) bacteria were detected on the sides and floors of lorries [10] and contaminated transporters were found to be responsible for spreading classical swine fever to different farms in Lithuania [11]. By moving animals with latent or asymptomatic infections this enables disease to spread to wherever the animal travels or where the necessary vectors may be present. Particularly in the case of epidemic diseases where the reduction of time from introduction of a hazard to its detection can enable early response and thereby lead to a reduction in intervention costs to contain an outbreak [12], effective surveillance is critical. Few surveillance systems however, are designed based on international livestock movement data, even though such data can provide information on the quantity and seasonality of livestock movements, the types of movement (for example flows from production of point of lay birds to laying units), the route the animals take and associated stopover or resting points. Surveillance for many livestock species occurs at the farm where it is the responsibility of the farmer (and veterinarian) to report notifiable diseases or at the abattoir where it is the role of the official veterinarian to inspect livestock according to Council Regulation (EC) 854/2004 [13] and report notifiable diseases to the national authorities, which in the UK is the Department of the Environment, Food and Rural Affairs (DEFRA), which in turn must inform the European Food Safety Authority (EFSA) as stated in Council Regulation (EC) 178/2002 [14].

Network analyses are useful ways of visualising the countries that are importing animals from a great number of other countries (high level of indegree) and countries that are exporting to a high number of countries (outdegree), these are values that can change temporally. They have been used to find out movement between farms of different species, for example, fish movement between farms in Scotland [15] and a study of pig and cattle movement between farms in Sweden [16]. Countries with a high indegree, which for the purposes of this study has a maximum number of 27 (the number of countries, i.e. (nodes, within this study and the EU as of 2011) that could be used to rank countries, can be more vulnerable to introducing disease due to importing animals from a greater number of countries than those with a low indegree whilst countries with a high outdegree may have a great ability to be able to transmit a disease to many countries; this highlights the importance of understanding levels of disease within trading countries. Information about the indegree and outdegree of farms was used by Frössling et al. (2012) [17] to investigate whether it could be used to target the surveillance of two cattle diseases in Sweden, based on a threshold of in- and out-degrees. They found a positive association between a positive test result and the purchase of animals and proposed approaches to design risk-based surveillance based on cattle movement data. Networks can also be used to quantify the proportion of international partners trading with each other (dyadic contacts) compared with the maximum number of national trading partners available for trade within an area allowing a comparison to be made between species and production systems [16]. The higher the density the more connected countries are with respect to the animal being traded and the more countries that may be at risk from contracting a disease from buying in infected livestock. A measure of mixing within a network is to look at its transitivity which indicates whether countries that a country is trading animals to are also trading animals with each other (a triad) [18]. The greater the level of transitivity the faster a disease can spread between countries and potentially infect many countries within the European area [19]. Transitivity and density for different communities of wild and domestic ungulates were investigated for the propensity to transmit *E. coli* by VanderWaal et al. (2014) [20]. However, the network may only consider the point of origin and destination and not necessarily consider the route itself that may involve briefly stopping in other countries where a disease transmission event may occur, for example FMD in France [9].

We hypothesise that the description of trade networks can inform the design of more efficient animal health surveillance systems that may enable a more rapid investigation or response to be implemented. Different species being transported for different purposes will have networks of different densities and different countries with the greatest indegree or outdegree. The aim of this project was to map live animal trade networks in EU countries and assess potential differences between species and purposes of transport. This was done by illustrating the number of live animal imports and exports between 27 EU countries including the number of country contacts and numbers of livestock units (LSU, a unit that takes into account the age, sex, purpose of animals with dairy cows having a reference number of 1) moved determining the density of networks and similarities of networks between species.

## Results

Table 1 illustrates the median livestock intra-community movements (expressed in livestock units) and the densities of the transport networks. By far the most heavily moved animal species within Europe in 2011 were poultry for slaughter and breeding, followed by poultry for ‘other’ purposes, pigs for fattening, pigs for slaughter and cattle for fattening; goats were the least traded species. Generally more LSUs were transported for fattening than for slaughter.

**Table 1:** **The median and interquartile range of livestock units (LSU) being transported within the Europe Union for different purposes, the density and transitivity of each transport network**

**Trade purpose**	**LSU median (IQR)**	**Density**	**Transitivity**
Cattle breeding	207 (34–947.5)	0.35	0.64
Cattle fattening	1267 (203–8283)	0.23	0.56
Cattle slaughter	647 (138.8-5381)	0.13	0.56
Cattle other	15 (3–115.5)	0.08	0.28
Pig breeding	86.5 (10–964)	0.24	0.62
Pig fattening	1762 (227.1-8923)	0.13	0.58
Pig slaughter	1433 (175.5-12260)	0.17	0.62
Pig other	7.8 (0.6-41.4)	0.06	0.36
Sheep breeding	6.1 (1.25-26.05)	0.16	0.47
Sheep fattening	130.7 (30–589.4)	0.11	0.44
Sheep slaughter	113.2 (32.33-564.2)	0.11	0.44
Sheep other	5.3 (0.35-46.75)	0.05	0.22
Goat breeding	1.8 (0.4-8.2)	0.09	0.34
Goat fattening	20.7 (3.25-53)	0.03	0.18
Goat slaughter	31.2 (5.1-255.2)	0.03	0.30
Goat other	0.4 (0.22-2.875)	0.03	0.25
Poultry breeding	2152 (376.6-13760)	0.24	0.55
Poultry slaughter	2645 (549.9-26530)	0.1	0.43
Poultry other	1570 (303.3-7353)	0.2	0.50
Equine breeding	4.4 (1.6-21)	0.17	0.49
Equine registered	9.6 (2.8-39.6)	0.45	0.67
Equine slaughter	145.6 (8.4-458)	0.05	0.31
Equine other	7.2 (1.6-37.2)	0.21	0.58

**Figure 1:**
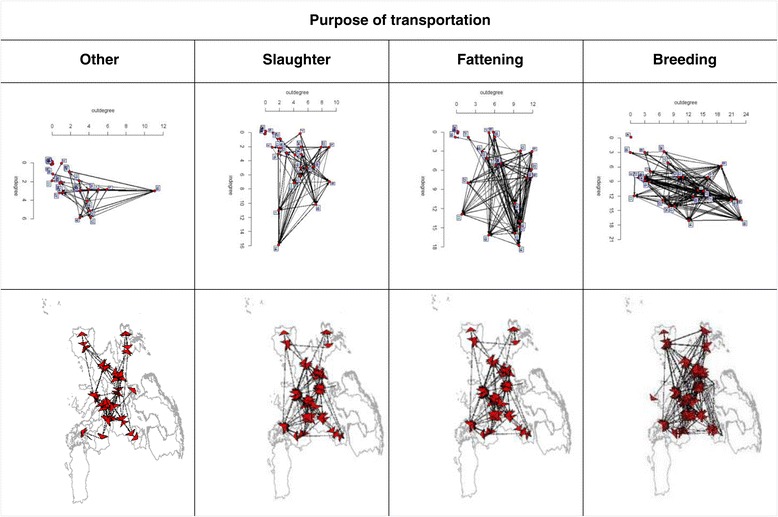
**The outdegree is shown against the indegree for the trade of cattle for different purposes on the left column of the table and the geographical movement across Europe is shown on the right column of the table.** The arrows between the countries indicate trade between the countries. The numbers in the figures refer to the corresponding countries: [1] Austria, [2] Belgium, [3] Bulgaria, [4] Cyprus, [5] Czech Republic, [6] Denmark, [7] Estonia, [8] Finland, [9] France, [10] Germany, [11] Greece, [12] Hungary, [13] Ireland, [14] Italy, [15] Lithuania, [16] Latvia, [17] Luxembourg, [18] Malta, [19] Netherlands, [20] Poland, [21] Portugal, [22] Romania, [23] Slovakia, [24] Slovenia, [25] Spain, [26] Sweden and [27] UK.

**Figure 2:**
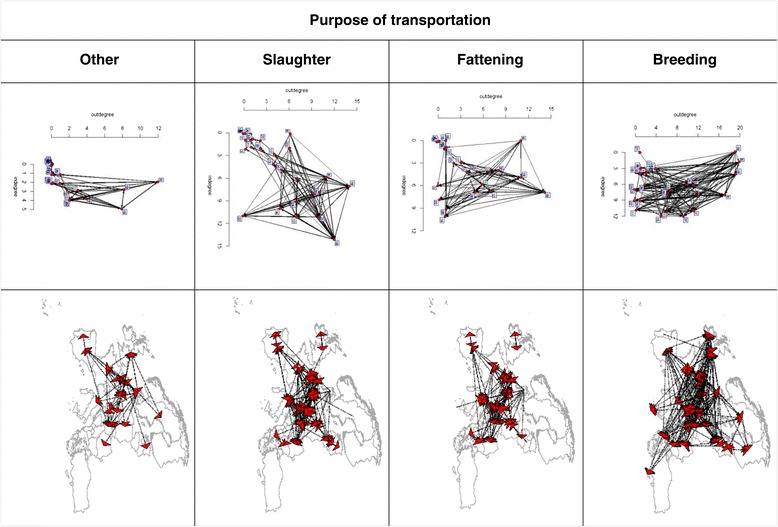
**The outdegree is shown against the indegree for the trade of pigs for different purposes on the left column of the table and the geographical movement across Europe is shown on the right column of the table.** The arrows between the countries indicate trade between the countries. The numbers in the figures refer to the corresponding countries: [1] Austria, [2] Belgium, [3] Bulgaria, [4] Cyprus, [5] Czech Republic, [6] Denmark, [7] Estonia, [8] Finland, [9] France, [10] Germany, [11] Greece, [12] Hungary, [13] Ireland, [14] Italy, [15] Lithuania, [16] Latvia, [17] Luxembourg, [18] Malta, [19] Netherlands, [20] Poland, [21] Portugal, [22] Romania, [23] Slovakia, [24] Slovenia, [25] Spain, [26] Sweden and [27] UK.

**Figure 3:**
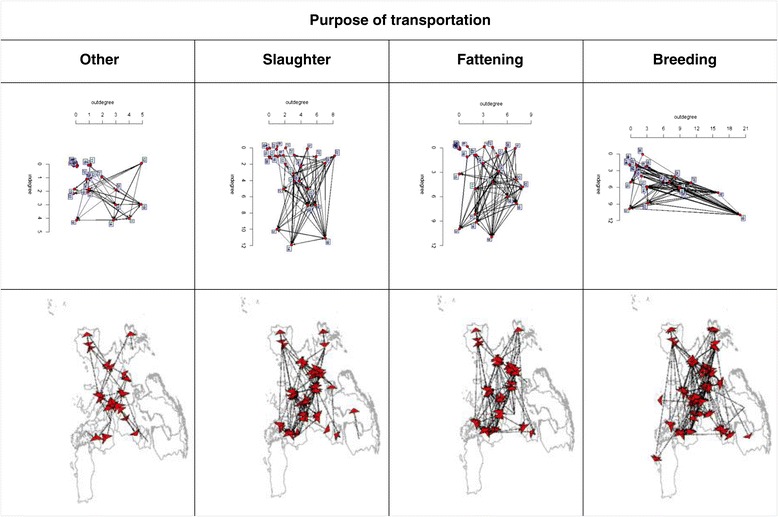
**The outdegree is shown against the indegree for the trade of sheep for different purposes on the left column of the table and the geographical movement across Europe is shown on the right column of the table.** The arrows between the countries indicate trade between the countries. The numbers in the figures refer to the corresponding countries: [1] Austria, [2] Belgium, [3] Bulgaria, [4] Cyprus, [5] Czech Republic, [6] Denmark, [7] Estonia, [8] Finland, [9] France, [10] Germany, [11] Greece, [12] Hungary, [13] Ireland, [14] Italy, [15] Lithuania, [16] Latvia, [17] Luxembourg, [18] Malta, [19] Netherlands, [20] Poland, [21] Portugal, [22] Romania, [23] Slovakia, [24] Slovenia, [25] Spain, [26] Sweden and [27] UK.

**Figure 4:**
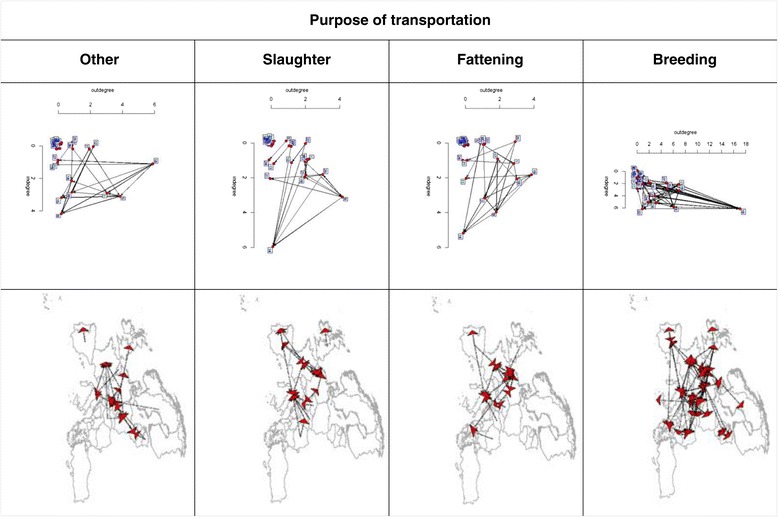
**The outdegree is shown against the indegree for the trade of goats for different purposes on the left column of the table and the geographical movement across Europe is shown on the right column of the table.** The arrows between the countries indicate trade between the countries. The numbers in the figures refer to the corresponding countries: [1] Austria, [2] Belgium, [3] Bulgaria, [4] Cyprus, [5] Czech Republic, [6] Denmark, [7] Estonia, [8] Finland, [9] France, [10] Germany, [11] Greece, [12] Hungary, [13] Ireland, [14] Italy, [15] Lithuania, [16] Latvia, [17] Luxembourg, [18] Malta, [19] Netherlands, [20] Poland, [21] Portugal, [22] Romania, [23] Slovakia, [24] Slovenia, [25] Spain, [26] Sweden and [27] UK.

**Figure 5:**
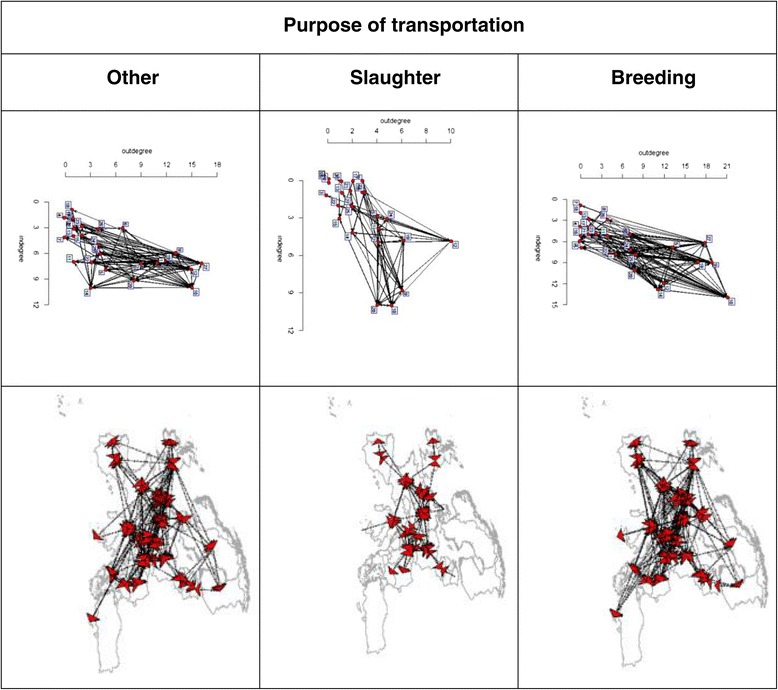
**The outdegree is shown against the indegree for the trade of poultry for different purposes on the left column of the table and the geographical movement across Europe is shown on the right column of the table.** The arrows between the countries indicate trade between the countries. The numbers in the figures refer to the corresponding countries: [1] Austria, [2] Belgium, [3] Bulgaria, [4] Cyprus, [5] Czech Republic, [6] Denmark, [7] Estonia, [8] Finland, [9] France, [10] Germany, [11] Greece, [12] Hungary, [13] Ireland, [14] Italy, [15] Lithuania, [16] Latvia, [17] Luxembourg, [18] Malta, [19] Netherlands, [20] Poland, [21] Portugal, [22] Romania, [23] Slovakia, [24] Slovenia, [25] Spain, [26] Sweden and [27] UK.

**Figure 6:**
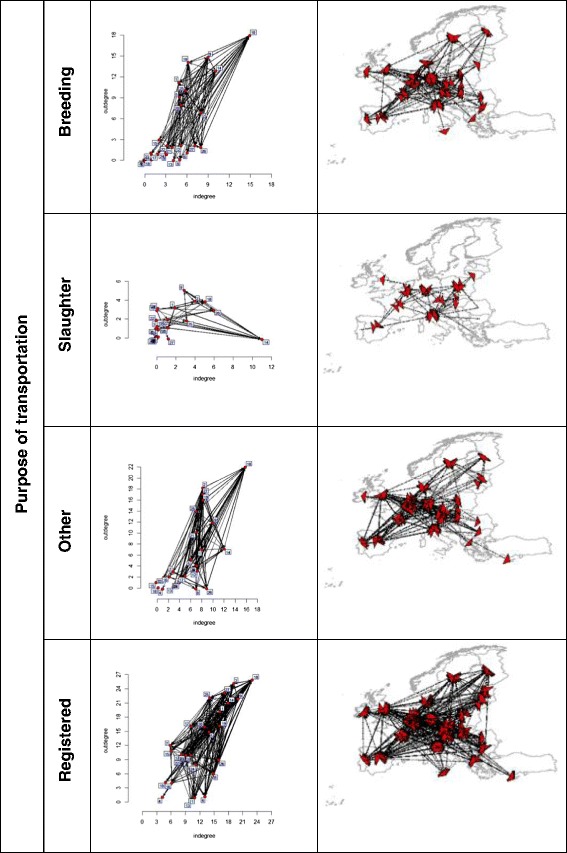
**The outdegree is shown against the indegree for the trade of equines for different purposes on the left column of the table and the geographical movement across Europe is shown on the right column of the table.** The arrows between the countries indicate trade between the countries. The numbers in the figures refer to the corresponding countries: [1] Austria, [2] Belgium, [3] Bulgaria, [4] Cyprus, [5] Czech Republic, [6] Denmark, [7] Estonia, [8] Finland, [9] France, [10] Germany, [11] Greece, [12] Hungary, [13] Ireland, [14] Italy, [15] Lithuania, [16] Latvia, [17] Luxembourg, [18] Malta, [19] Netherlands, [20] Poland, [21] Portugal, [22] Romania, [23] Slovakia, [24] Slovenia, [25] Spain, [26] Sweden and [27] UK.

The density of movement (Table 1) shows that there was greater connectivity for cattle than for the heavily traded poultry. Breeding networks were found to be denser than those for other purposes. This may be due to the number of consignments needed to move the relative units of animals. The geographical trade flows are shown in Figures 1, 2, 3, 4, 5 and 6. The transitivity indicates that disease would spread more slowly for ‘other’ purposes of animal movement than for breeding, fattening or slaughter with the exception of poultry and equines.

Figures 1, 2, 3, 4, 5 and 6 show the in- and outdegrees of livestock unit movements in the EU on the left and the geographical trade flows in the right, which are separated by species and by purpose of trade. The axes of the graphs of the in- and outdegrees reflect the numbers of trading partners. The countries in the top right received and exported animals with the greatest number of countries, whilst the bottom left indicates those that have little or no export or import trade with other countries. Some countries are found in the top right corner with regards to many different animal movements e.g. Germany, whilst others rarely buy or sell to the other 26 countries considered in this study e.g. Cyprus, Finland and Sweden, whilst other countries import from many countries and export to few e.g. Italy.

Very few shipments of weaned cattle, sheep and goats require a rest period of 24 hours (Additional file 1), whereas many unweaned animals would require a 24 hour break in their journey from their point of origin to their final destination (Additional file 2) as would most journeys of poultry 72 hours after hatching (Additional file 3).

The proportion of national populations imported and exported could be calculated for all species except equines for which there was no data for the year 2011 (Additional file 4). Goats were the species where imports and exports were a low proportion of the national population in comparison with pigs where many countries were importing and exporting high proportions of their national population, the officially recorded number of animals of that species in the particular country.

## Discussion

The poultry and pig sectors had the greatest number of LSU movements, which are being used to indicate the potential opportunities of pathogen introduction and spread, implying that they require more attention in terms of disease prevention and management, while the equine and goat sectors had the greatest and lowest densities of movements respectively. In addition to LSU movements larger proportions of national pig populations are imported compared with species such as goats increasing the possibility for the introduction of infected animals to an existing population. For poultry, the highest numbers of LSUs moved were for slaughter, which may present less of a risk of introducing disease to an existing population, as the animals are likely to be transported from the production site directly to the slaughter point. However, many poultry journeys would require a break in transit emphasising the vulnerability of the chain and need for adequate surveillance. Poultry for breeding had the second highest LSU movements overall, which likely reflects the current structure of commercial poultry production. Pure line grandparent and parent stock for breeding are produced by only a limited number of breeding organisations worldwide. For example, the two companies Aviagen and Cobb, have a market share of more than 85% of the commercial broilers produced in the EU and use their global network of distributors to serve almost all European countries [21]. The breeder farms supplied with young breeding stock have links to hatcheries that produce day old chicks, broiler or layer farms, and slaughterhouses. This system leads to transport of young breeders, hatching eggs and day old chicks. In pigs, heavy movements were recorded for fattening, which reflects ongoing changes in production centres in the EU. In fact, more than two thirds of breeding pigs are produced in Denmark, Germany, Spain, France, the Netherlands and Poland with half of the breeding pigs at regional level being concentrated in eleven regions in these six countries [22]. Germany is the main importer of fattening pigs, with an indegree of 7 and Denmark is the main exporter with an outdegree of 11. Moreover, pigs for breeding and fattening as well as poultry for breeding were shown to have among the highest transitivities, indicating that disease spread in these networks would be fast if uncontained. Hence, solely taking into account trade data, surveillance efforts would need to focus on poultry for breeding and pigs for breeding and fattening. However, a mapping of surveillance in seven European countries showed that the highest proportion of surveillance components in place were for cattle [23]. Similarly, a recent literature review on animal health issues (including zoonoses) researched in the EU showed that cattle and buffalo were the species most frequently studied in the EU [24]; this may reflect differences in resource allocation for surveillance and disease mitigation. The reasons for this may be that cattle harbour or are perceived to harbour more pathogens than other species, that outbreaks in cattle systems have higher impact, that cattle receives more attention than other species for cultural or historical reasons, or that disease prevention and management in cattle systems are of lower quality. Currently, there are no multi-pathogen, multi-species systematic risk assessments available at EU level that would allow a comparison of these factors. Breeding networks were found to be more highly connected with more trade between countries indicating disease may spread more easily through them. This is of concern as these animals are not intended to be slaughtered on arrival and will produce new animals, therefore stringent precautions are needed to protect these populations, particularly if they are diseases not covered by EU legislation, for example the diseases listed in Council Regulation (EC) 722/2013 [25]. The density of international agri-trade calculated by Ercsey-Ravasz et al. (2012) [26] was 0.33 which was comparable with density of many networks in this study. However, in national networks the densities and transitivities are smaller, which are due to the greater number of farms involved in national animal production compared with the number of countries involved in this study. The cattle trade network in France had a very low annual level of transitivity indicating that disease spread would be slower than that between European countries [27]. The pig and cattle networks in Sweden had lower transitivities than international networks of these species [16] as did the transitivity of pig movements in Denmark [28] and the UK [29].

The location of countries in Figures 1, 2, 3, 4, 5 and 6 gave an indication of where surveillance could be targeted with countries in the upper right quadrant both importing and exporting high numbers of LSU, which means that they need to monitor both production to export healthy animals and import processes to avoid introduction of disease. Countries in the lower right quadrant may need to consider strengthening surveillance related to import processes. Many national studies have found that the majority of animal movements are between premises with lower indegrees and outdegrees as shown in a study by Smith et al. 2013 [29], this reduces the likelihood of disease transmission to many different areas, reducing the level of surveillance needed. Many countries trading cattle were found to have an in or out degree equal or greater than five. This was the threshold that was calculated to require enhanced surveillance for bovine coronavirus in a study on trade and cattle in Sweden by Frössling et al. 2012 [17]. Consequently, there seems to be ample opportunity to take advantage of trade network data to enhance surveillance. The evolution of trade networks over time at the EU level could be monitored using indegrees, outdegrees, and transitivity. Such monitoring would provide information at the systems level and allow observations of changes in networks over time and where consequent surveillance efforts should be focused. Higher-level surveillance capturing trends or changes in trade patterns could complement existing surveillance systems that are commonly disease centered. The differences across countries in terms of indegrees and outdegrees also bring up the question of who has the responsibility for disease control, including surveillance – the buyer, the seller or relevant food business operator depending on the stage of livestock production [30]. While the draft new EU Animal Health Law [31] refers to listed diseases and pre-dominantly supports disease centered surveillance, it also creates a framework for the better use of the synergies between surveillance undertaken by the different actors in the field to ensure the most effective and cost efficient use of surveillance resources as well as promotion of data availability and facilitation of data exchange.

Transportation itself is stressful for animals as indicated in many studies in many species for example cortisol in pigs [32]; heart rate and cortisol in cattle [33]; cortisol in lambs [34]; cortisol in horses [35]; increasing susceptibility to disease and may enhance the likelihood of shedding pathogenic agents in transit or in the receiving country, which may lead to infection in other animals. It is common to refer to malaise post-transportation as shipping illness [36]. However, pathogens may be introduced or spread from transporters and not just from the animals that they transport. Studies have demonstrated that transporters need to be thoroughly cleaned to prevent them from acting as a source of pathogens to subsequently carried animals, for example to prevent transmission of porcine reproductive and respiratory syndrome virus, that can survive in transporters, being transferred to pigs [37]. Rest stops are infrequent for some species, however, if animals from more than one origin are rested in the same place it may allow for disease spread. This is most likely to impact animals traded for breeding and fattening purposes that have more LSUs and are more highly connected than animals already at slaughter weight. These are animals that will live in the receiving country for a period of time that may enable pathogen transfer. Many of the highly connected countries (with high in and out degrees in the top right of Figures 1, 2, 3, 4, 5 and 6) for example Germany are geographically located in an area (Central Europe) that minimises the distances and therefore time that animals have to travel reducing the need for rest breaks and the consequent potential for pathogen transfer. Many of the long distances are from countries that rarely trade with mainland Europe for example Cyprus. Many animals undergo long journeys between countries. The time in transit is a concern with regards of the potential for disease to spread along trade routes [9]. This has implications for policy around the planning of livestock production and slaughter. Ideally, large production facilities would not be placed adjacent to well-known and used trade routes and or resting points. However, such information is only of use to policy makers if it is captured in a systematic and continuous way allowing to monitor trends, change and modify policies accordingly if deemed necessary.

### Limitations

The analyses have only considered the spatial aspect of trade and not taken into account temporal variations that may occur altering the relationships between the countries (nodes) and the respective network, and affect the likelihood of an animal being infectious with a disease. Animal populations fluctuate within a year and the population recorded in December was used to calculate the proportion of animals being imported or exported into a country, therefore it may have under or overestimated the actual population at the time of movement. For example the majority of lambs are born between January and April increasing the sheep population until they reach slaughter weight and are culled, which occurs before December. Networks are highly dynamic and these changes in movements between countries will need to be considered by surveillance programs using this approach. One method that may address this is to use exponential random graph models that can incorporate a range of different distributions of connectivity between the nodes to create many different networks, which can be compared with the data to find a model that best fits the current trade pattern [38].

The distances that animals are transported between countries may be shorter or longer than the distances between centroids. In addition, there are many different routes across Europe that may be used and this may be worth investigating in future analyses with regards to distance, time and mixing between countries. This means that our calculations for whether particular species need a rest break for movement between particular countries are generalised so that there may be fewer or greater numbers of animals being rested en-route to their destination country altering the potential for pathogen exposure.

The analyses did not take into account the numbers of convoys or animals and the mixing of animals: from different farms per convoy, at resting places, at borders, when received by individuals and at markets in the country of destination. These factors will have an impact on contact between potentially naïve and infectious animals, pathogen exposure and susceptibility.

The analyses could not take into animals being bought and sold on to more than one country i.e. the chain of infection [16] and assumed that an animal moved once between countries in its lifetime.

## Conclusions

Creating networks has enabled us to visualise the countries that have a higher level of involvement in animal trade. Using network analysis we were able to determine the extent to which a disease may spread, the production systems where disease spread may be more rapid, for example registered horses and breeding cattle, pigs and poultry, and facilitates comparisons with networks in other areas. Similarities between countries, species and production purposes has the potential to inform international surveillance policies that take into account trade patterns. The study has highlighted the vulnerability of the pig network to disease, which is of increasing concern due to the proximity of African Swine Fever to the EU and the potential for wildlife to introduce the disease [11]. This information could complement the national movement recording systems that are mandatory for cattle throughout the EU [39] that will soon be implemented in sheep and goats now that their form of identification tags have been decided upon [40], and being planned for porcines [41] to produce a more robust surveillance plan.

## Methods

Data on numbers of live cattle, goats, horses, pigs, poultry and sheep movements in 27 EU countries were obtained from Directorate General Sanco Animal Health DG Sanco unit G2 activity report for the year 2011 obtained from http://ec.europa.eu/food/animal/resources/publications_en.htm. The data obtained related to the production purpose of the animals, which fell into five categories: breeding, fattening, slaughter, registered and other (e.g. pets, show animals). These categories were analysed separately and combined for each species.

The numbers of animals were converted into livestock units to enable comparison between species using the following conversion factors derived from the Eurostat glossary on statistics (2013) [42]: pigs 0.5 (breeding), pigs 0.3 (other), goats 0.1, sheep 0.1, horses 0.8 and poultry 0.014. All data were obtained at a national level from publically accessible databases and no animal experimentation occurred nor consultation with animal owners therefore ethical approval was not needed.

All the analyses and associated network figures were created and carried out using R 3.0.1. [43]. Networks were created from adjacency matrices and their densities were calculated using network function found in R package Network [44]. The in and out degrees were calculated and respective graphs were produced using the degree and network.layout.degree functions in R package Network [44]. The transitivity of each network was calculated using the gtrans function in the SNA package [45]. Trade maps in the Figures 1, 2, 3, 4, 5 and 6 were produced by merging shapefiles of all the countries of Europe downloaded from maplibrary.org (www.gadm.org/, 2010, gadm version 9) into one polygon (Europe) using ArcGIS 10.1 [46]. The map of Europe was then read into R using the function readShapePoly found in the Maptools package [47]. Centroids (the co-ordinates for the centre of a country) were calculated for each country and linked with respective importing and exporting countries were calculated using the calcCentroid function in R package PBSmapping [48]. Curved lines and arrows were drawn between the centroids for each movement using the gcIntermediate function found in the geosphere package [49].

To be able to relate the numbers of animals being traded with the animal populations of the countries, the numbers of animals of each species were obtained for 2011 from the Eurostat database. The data used was for December as this was the only calendar month available for all species. A movement:standing population ratio was calculated for both animal imports and exports through adding the total number of breeding, fattening, slaughter, registered and other animals being moved and dividing by the total population of animals of that species in the exporting or importing country.

To illustrate the number of animal journeys that require 24 hour rest periods during transit, distances that animals would have to travel were approximated by estimating arc distances from one capital city to the other using www.timeanddate.com. The time in transit before animals are required to have a 24 hour rest period were obtained from Council Regulation EC 1/2005 [4]. The Regulation states that unweaned cattle, goats, sheep, pigs and horses require a 24 hour rest period after 18 hours of travel. Weaned cattle, goats and sheep can be in transit for 28 hours without a rest, whereas weaned pigs and domestic horses need to be rested after 24 hours of transportation. Any animal being transported by boat should be rested for 12 hours at the port after being unloaded. The law for poultry and rabbits states that they can travel for up to 12 hours without food or water and whereas chicks within 72 hours of hatching can travel for up to 24 hours without food or water. To gauge whether a journey between two rest points would need a break the following equation was used given the assumption that a vehicle would be travelling at an average 80 kilometres an hour.

$$ 24\kern0.5em \mathrm{hour}\kern0.5em \mathrm{rest}\kern0.5em \mathrm{period}\kern0.5em =\kern0.5em \frac{Distance\kern0.5em  between\kern0.5em  cities}{Duration\kern0.5em  of\kern0.5em  travel\kern0.5em  before\kern0.5em  24\kern0.5em  hours\kern0.5em  rest\kern0.5em  period\kern0.5em * 80\kern0.5em  km/h} $$

## Additional files

Additional file 1:
**Journeys that would require rest breaks due to being over 28 hours long or over 24 hours long.** These data are displayed in tables. Additional file 2:
**Journeys that would require rest breaks for unweaned animals.** The data are displayed in a table. Additional file 3:
**Journeys that would require rest breaks for poultry other than chicks <72 hours old.** The data are displayed in a table. Additional file 4:
**The proportions of national animal imports and exports compared with the national population.** These data are displayed in separate tables for each species.

## Abbreviations

AI: Avian influenza
BSE: Bovine spongiform encephalopathy
BTV: Bluetongue virus
EU: European Union
FAO: Food and Agricultural Organisation of the United Nations
FMD: Foot and mouth disease
IQR: Interquartile range
LSU: Livestock units
SBV: Schmallenberg virus
TRACES: Trade Control and Expert System
