# Supplementary material for: Livestock trade networks for guiding animal health surveillance
Source: BMC Vet Res. 2015 Apr 1;11:82. doi: 10.1186/s12917-015-0354-4 (PMC4411738; doi:10.1186/s12917-015-0354-4)
Supplement: Additional file 1: — Journeys that would require rest breaks due to being over 28 hours long or over 24 hours long. These data are displayed in tables. [file 12917_2015_354_MOESM1_ESM.pdf]

Additional file 1.

Table 1. The distances between European capital cities with highlighted distances indicating journeys of over 28 hours (weaned cattle, sheep and goats) that would require a rest period. Numbers in bold italics indicates countries where animals would be transported by sea and need to be rested on arrival at the destination port.

Countries	Austria	Belgium	Bulgaria	Cyprus	Czech Republic	Denmark	Estonia	Finland	France	Germany	Greece	Hungary	Ireland	Italy	Lithuania	Latvia	Luxembourg	Malta	Netherlands	Poland	Portugal	Romania	Slovakia	Slovenia	Spain	Sweden
Belgium	917	0																								
Bulgaria	819	1701	0																							
Cyprus	***2016***	***2906***	***1205***	***0***																						
Czech Republic	252	719	1068	***2258***	0																					
Denmark	870	767	1638	***2777***	634	0																				
Estonia	1363	1603	1865	***2770***	1232	839	0																			
Finland	1440	1652	1947	***2845***	1304	885	82	0																		
France	1037	266	1762	***2955***	886	1030	1864	1914	0																	
Germany	523	651	1320	***2492***	280	356	1045	1109	879	0																
Greece	1284	2092	525	***916***	1536	2138	2388	2469	2100	1804	0															
Hungary	217	1133	630	***1812***	446	1014	1381	1462	1250	691	1124	0														
Ireland	***1687***	***778***	***2479***	***3684***	***1469***	***1243***	***2010***	***2031***	***782***	***1320***	***2860***	***1902***	***0***													
Italy	766	1174	898	***1961***	923	1533	2127	2204	1107	1183	1054	811	***1889***	0												
Lithuania	948	1470	1340	***2256***	898	816	531	612	1702	823	1860	910	***2056***	1704	0											
Latvia	1103	1458	1586	***2519***	996	727	280	326	1709	848	2109	1106	***1961***	1869	263	0										
Luxembourg	765	187	1529	***2732***	598	802	1617	1673	290	601	1909	981	***954***	989	1421	1443	0									
Malta	***1377***	***1850***	***1070***	***1709***	***1576***	***2203***	***2720***	***2800***	***1749***	***1849***	***852***	***1342***	***2526***	***689***	***2246***	***2448***	***1669***	***0***								
Netherlands	938	172	1747	***2950***	712	623	1461	1506	432	577	2167	1150	***759***	1298	1371	1335	319	***1982***	0							
Poland	557	1163	1076	***2136***	519	671	834	914	1372	520	1600	547	***1832***	1318	392	560	1083	***1888***	1096	0						
Portugal	2303	1715	2761	***3772***	2248	2482	3317	3367	1454	2315	2859	2476	***1642***	1920	3127	3157	1715	***2114***	1866	2765	0					
Romania	858	1774	296	***1201***	1082	1576	1672	1752	1876	1297	744	643	***2544***	1141	1141	1399	1617	***1366***	1791	947	2982	0				
Slovakia	56	971	776	***1969***	292	894	1351	1430	1094	554	1251	162	***1739***	785	921	1086	821	***1378***	988	534	2355	805	0			
Slovenia	279	921	794	***1992***	449	1080	1638	1714	969	724	1176	382	***1696***	490	1225	1380	742	***1127***	991	835	2102	926	306	0		
Spain	1813	1317	2259	***3291***	1775	2075	2898	2953	1052	1870	2375	1979	***1450***	1367	2666	2717	1281	***1670***	1482	2294	504	2479	1863	1602	0	
Sweden	1242	1283	1885	***2908***	1055	523	380	397	1548	813	2409	1319	***1634***	1978	678	443	1325	***2618***	1128	809	2994	1744	1246	1497	2596	0
UK	***1237***	***321***	***2018***	***3221***	***1035***	***958***	***1788***	***1826***	***343***	***932***	***2394***	***1454***	***466***	***1434***	***1728***	***1681***	***490***	***2088***	***358***	***1452***	***1586***	***2095***	***1291***	***1231***	***1263***	***1437***

Table 2. The distances between European capital cities, with those in bold indicating journeys of over 24 hours (pigs, domestic horses and chickens as long as the journey is completed within 72 hours of hatching) that would require a rest period. Numbers in bold italics indicates countries where animals would be transported by sea and need to be rested on arrival at the destination port.

Countries	Austria	Belgium	Bulgaria	Cyprus	Czech Republic	Denmark	Estonia	Finland	France	Germany	Greece	Hungary	Ireland	Italy	Lithuania	Latvia	Luxembourg	Malta	Netherlands	Poland	Portugal	Romania	Slovakia	Slovenia	Spain	Sweden
Belgium	917	0																								
Bulgaria	819	1701	0																							
Cyprus	***2016***	***2906***	***1205***	***0***																						
Czech Republic	252	719	1068	***2258***	0																					
Denmark	870	767	1638	***2777***	634	0																				
Estonia	1363	1603	1865	***2770***	1232	839	0																			
Finland	1440	1652	**1947**	***2845***	1304	885	82	0																		
France	1037	266	1762	***2955***	886	1030	1864	1914	0																	
Germany	523	651	1320	***2492***	280	356	1045	1109	879	0																
Greece	1284	2092	525	***916***	1536	**2138**	**2388**	**2469**	**2100**	1804	0															
Hungary	217	1133	630	***1812***	446	1014	1381	1462	1250	691	1124	0														
Ireland	***1687***	***778***	***2479***	***3684***	***1469***	***1243***	***2010***	***2031***	***782***	***1320***	***2860***	***1902***	***0***													
Italy	766	1174	898	***1961***	923	1533	**2127**	**2204**	1107	1183	1054	811	***1889***	0												
Lithuania	948	1470	1340	***2256***	898	816	531	612	1702	823	1860	910	***2056***	1704	0											
Latvia	1103	1458	1586	***2519***	996	727	280	326	1709	848	**2109**	1106	***1961***	1869	263	0										
Luxembourg	765	187	1529	***2732***	598	802	1617	1673	290	601	1909	981	***954***	989	1421	1443	0									
Malta	***1377***	***1850***	***1070***	***1709***	***1576***	***2203***	***2720***	***2800***	***1749***	***1849***	***852***	***1342***	***2526***	***689***	***2246***	***2448***	***1669***	***0***								
Netherlands	938	172	1747	***2950***	712	623	1461	1506	432	577	2167	1150	***759***	1298	1371	1335	319	***1982***	0							
Poland	557	1163	1076	***2136***	519	671	834	914	1372	520	1600	547	***1832***	1318	392	560	1083	***1888***	1096	0						
Portugal	**2303**	1715	**2761**	***3772***	**2248**	**2482**	**3317**	**3367**	1454	**2315**	**2859**	**2476**	***1642***	1920	**3127**	**3157**	1715	***2114***	1866	**2765**	0					
Romania	858	1774	296	***1201***	1082	1576	1672	1752	1876	1297	744	643	***2544***	1141	1141	1399	1617	***1366***	1791	947	**2982**	0				
Slovakia	56	971	776	***1969***	292	894	1351	1430	1094	554	1251	162	***1739***	785	921	1086	821	***1378***	988	534	**2355**	805	0			
Slovenia	279	921	794	***1992***	449	1080	1638	1714	969	724	1176	382	***1696***	490	1225	1380	742	***1127***	991	835	**2102**	926	306	0		
Spain	1813	1317	**2259**	***3291***	1775	**2075**	**2898**	**2953**	1052	1870	**2375**	**1979**	***1450***	1367	**2666**	**2717**	1281	***1670***	1482	**2294**	504	**2479**	1863	1602	0	
Sweden	1242	1283	1885	***2908***	1055	523	380	397	1548	813	**2409**	1319	***1634***	**1978**	678	443	1325	***2618***	1128	809	**2994**	1744	1246	1497	**2596**	0
UK	***1237***	***321***	***2018***	***3221***	***1035***	***958***	***1788***	***1826***	***343***	***932***	***2394***	***1454***	***466***	***1434***	***1728***	***1681***	***490***	***2088***	***358***	***1452***	***1586***	***2095***	***1291***	***1231***	***1263***	***1437***
