# Supplementary material for: Livestock trade networks for guiding animal health surveillance
Source: BMC Vet Res. 2015 Apr 1;11:82. doi: 10.1186/s12917-015-0354-4 (PMC4411738; doi:10.1186/s12917-015-0354-4)
Supplement: Additional file 2: — Journeys that would require rest breaks for unweaned animals. The data are displayed in a table. [file 12917_2015_354_MOESM2_ESM.pdf]

**Additional file 2.**

**Table 1. The distances between European capital cities, with those in bold indicating journeys of over 18 hours (unweaned animals) that would require a rest period. Numbers in bold italics indicates countries where animals would be transported by sea and need to be rested on arrival at the destination port.**

| Countries | Austria | Belgium | Bulgaria | Cyprus | Czech Republic | Denmark | Estonia | Finland | France | Germany | Greece | Hungary | Ireland | Italy | Lithuania | Latvia | Luxembourg | Malta | Netherlands | Poland | Portugal | Romania | Slovakia | Slovenia | Spain | Sweden |
|---|---|---|---|---|---|---|---|---|---|---|---|---|---|---|---|---|---|---|---|---|---|---|---|---|---|---|
| Belgium | 917 | 0 | | | | | | | | | | | | | | | | | | | | | | | | |
| Bulgaria | 819 | **1701** | 0 | | | | | | | | | | | | | | | | | | | | | | | |
| Cyprus | ***2016*** | ***2906*** | ***1205*** | ***0*** | | | | | | | | | | | | | | | | | | | | | | |
| Czech Republic | 252 | 719 | 1068 | ***2258*** | 0 | | | | | | | | | | | | | | | | | | | | | |
| Denmark | 870 | 767 | **1638** | ***2777*** | 634 | 0 | | | | | | | | | | | | | | | | | | | | |
| Estonia | 1363 | **1603** | **1865** | ***2770*** | 1232 | 839 | 0 | | | | | | | | | | | | | | | | | | | |
| Finland | 1440 | **1652** | **1947** | ***2845*** | 1304 | 885 | 82 | 0 | | | | | | | | | | | | | | | | | | |
| France | 1037 | 266 | **1762** | ***2955*** | 886 | 1030 | **1864** | **1914** | 0 | | | | | | | | | | | | | | | | | |
| Germany | 523 | 651 | 1320 | ***2492*** | 280 | 356 | 1045 | 1109 | 879 | 0 | | | | | | | | | | | | | | | | |
| Greece | 1284 | **2092** | 525 | ***916*** | **1536** | **2138** | **2388** | **2469** | **2100** | **1804** | 0 | | | | | | | | | | | | | | | |
| Hungary | 217 | 1133 | 630 | ***1812*** | 446 | 1014 | 1381 | 1462 | 1250 | 691 | 1124 | 0 | | | | | | | | | | | | | | |
| Ireland | ***1687*** | ***778*** | ***2479*** | ***3684*** | ***1469*** | ***1243*** | ***2010*** | ***2031*** | ***782*** | ***1320*** | ***2860*** | ***1902*** | ***0*** | | | | | | | | | | | | | |
| Italy | 766 | 1174 | 898 | ***1961*** | 923 | **1533** | **2127** | **2204** | 1107 | 1183 | 1054 | 811 | ***1889*** | 0 | | | | | | | | | | | | |
| Lithuania | 948 | **1470** | 1340 | ***2256*** | 898 | 816 | 531 | 612 | **1702** | 823 | 1860 | 910 | ***2056*** | 1704 | 0 | | | | | | | | | | | |
| Latvia | 1103 | **1458** | **1586** | ***2519*** | 996 | 727 | 280 | 326 | **1709** | 848 | **2109** | 1106 | ***1961*** | 1869 | 263 | 0 | | | | | | | | | | |
| Luxembourg | 765 | 187 | **1529** | ***2732*** | 598 | 802 | **1617** | **1673** | 290 | 601 | **1909** | 981 | ***954*** | 989 | 1421 | 1443 | 0 | | | | | | | | | |
| Malta | ***1377*** | ***1850*** | ***1070*** | ***1709*** | ***1576*** | ***2203*** | ***2720*** | ***2800*** | ***1749*** | ***1849*** | ***852*** | ***1342*** | ***2526*** | ***689*** | ***2246*** | ***2448*** | ***1669*** | ***0*** | | | | | | | | |
| Netherlands | 938 | 172 | **1747** | ***2950*** | 712 | 623 | **1461** | **1506** | 432 | 577 | **2167** | 1150 | ***759*** | 1298 | 1371 | 1335 | 319 | ***1982*** | 0 | | | | | | | |
| Poland | 557 | 1163 | 1076 | ***2136*** | 519 | 671 | 834 | 914 | 1372 | 520 | **1600** | 547 | ***1832*** | 1318 | 392 | 560 | 1083 | ***1888*** | 1096 | 0 | | | | | | |
| Portugal | **2303** | **1715** | **2761** | ***3772*** | **2248** | **2482** | **3317** | **3367** | **1454** | **2315** | **2859** | **2476** | ***1642*** | **1920** | **3127** | **3157** | **1715** | ***2114*** | **1866** | **2765** | 0 | | | | | |
| Romania | 858 | **1774** | 296 | ***1201*** | 1082 | **1576** | **1672** | **1752** | **1876** | 1297 | 744 | 643 | ***2544*** | 1141 | 1141 | 1399 | 1617 | ***1366*** | 1791 | 947 | **2982** | 0 | | | | |
| Slovakia | 56 | 971 | 776 | ***1969*** | 292 | 894 | 1351 | 1430 | 1094 | 554 | 1251 | 162 | ***1739*** | 785 | 921 | 1086 | 821 | ***1378*** | 988 | 534 | **2355** | 805 | 0 | | | |
| Slovenia | 279 | 921 | 794 | ***1992*** | 449 | 1080 | 1638 | 1714 | 969 | 724 | 1176 | 382 | ***1696*** | 490 | 1225 | 1380 | 742 | ***1127*** | 991 | 835 | **2102** | 926 | 306 | 0 | | |
| Spain | **1813** | 1317 | **2259** | ***3291*** | **1775** | **2075** | **2898** | **2953** | 1052 | **1870** | **2375** | **1979** | ***1450*** | 1367 | **2666** | **2717** | 1281 | ***1670*** | **1482** | **2294** | 504 | **2479** | **1863** | **1602** | 0 | |
| Sweden | 1242 | 1283 | **1885** | ***2908*** | 1055 | 523 | 380 | 397 | **1548** | 813 | **2409** | 1319 | ***1634*** | **1978** | 678 | 443 | 1325 | ***2618*** | 1128 | 809 | **2994** | **1744** | 1246 | **1497** | **2596** | 0 |
| UK | ***1237*** | ***321*** | ***2018*** | ***3221*** | ***1035*** | ***958*** | ***1788*** | ***1826*** | ***343*** | ***932*** | ***2394*** | ***1454*** | ***466*** | ***1434*** | ***1728*** | ***1681*** | ***490*** | ***2088*** | ***358*** | ***1452*** | ***1586*** | ***2095*** | ***1291*** | ***1231*** | ***1263*** | ***1437*** |
