# Supplementary material for: Livestock trade networks for guiding animal health surveillance
Source: BMC Vet Res. 2015 Apr 1;11:82. doi: 10.1186/s12917-015-0354-4 (PMC4411738; doi:10.1186/s12917-015-0354-4)
Supplement: Additional file 3: — Journeys that would require rest breaks for poultry other than chicks <72 hours old. The data are displayed in a table. [file 12917_2015_354_MOESM3_ESM.pdf]

Additional file 3.

Table 1. The distances between European capital cities, with those in bold indicating journeys of over 12 hours (Poultry other than chicks within 72 hours of hatching) that would require a rest period. Numbers in bold italics indicates countries where animals would be transported by sea and need to be rested on arrival at the destination port.

Countries	Austria	Belgium	Bulgaria	Cyprus	Czech_Republic	Denmark	Estonia	Finland	France	Germany	Greece	Hungary	Ireland	Italy	Lithuania	Latvia	Luxembourg	Malta	Netherlands	Poland	Portugal	Romania	Slovakia	Slovenia	Spain	Sweden
Austria	0																									
Belgium	917	0																								
Bulgaria	819	**1701**	0																							
Cyprus	***2016***	***2906***	***1205***	0																						
Czech_Republic	252	719	**1068**	**2258**	0																					
Denmark	870	767	**1638**	**2777**	634	0																				
Estonia	**1363**	**1603**	**1865**	**2770**	**1232**	839	0																			
Finland	**1440**	**1652**	**1947**	**2845**	**1304**	885	82	0																		
France	**1037**	266	**1762**	**2955**	886	**1030**	**1864**	**1914**	0																	
Germany	523	651	**1320**	**2492**	280	356	**1045**	**1109**	879	0																
Greece	**1284**	**2092**	525	916	**1536**	**2138**	**2388**	**2469**	**2100**	**1804**	0															
Hungary	217	**1133**	630	**1812**	446	**1014**	**1381**	**1462**	**1250**	691	1124	0														
Ireland	***1687***	***778***	***2479***	***3684***	***1469***	***1243***	***2010***	***2031***	***782***	***1320***	***2860***	***1902***	0													
Italy	766	**1174**	898	**1961**	923	**1533**	**2127**	**2204**	**1107**	**1183**	**1054**	**811**	**1889**	0												
Lithuania	948	**1470**	**1340**	**2256**	898	816	531	612	**1702**	823	**1860**	910	**2056**	**1704**	0											
Latvia	**1103**	**1458**	**1586**	**2519**	**996**	727	280	326	**1709**	848	**2109**	**1106**	**1961**	**1869**	263	0										
Luxembourg	765	187	**1529**	**2732**	598	802	**1617**	**1673**	290	601	**1909**	**981**	954	**989**	**1421**	**1443**	0									
Malta	***1377***	***1850***	***1070***	***1709***	***1576***	***2203***	***2720***	***2800***	***1749***	***1849***	***852***	***1342***	***2526***	***689***	***2246***	***2448***	***1669***	0								
Netherlands	938	172	**1747**	**2950**	712	623	**1461**	**1506**	432	577	**2167**	**1150**	759	**1298**	**1371**	**1335**	319	**1982**	0							
Poland	557	**1163**	**1076**	**2136**	519	671	834	914	**1372**	520	**1600**	547	**1832**	**1318**	392	560	**1083**	**1888**	**1096**	0						
Portugal	**2303**	**1715**	**2761**	**3772**	**2248**	**2482**	**3317**	**3367**	**1454**	**2315**	**2859**	2476	**1642**	**1920**	**3127**	**3157**	**1715**	**2114**	**1866**	**2765**	0					
Romania	858	**1774**	296	**1201**	**1082**	**1576**	**1672**	**1752**	**1876**	**1297**	744	643	**2544**	**1141**	**1141**	**1399**	**1617**	**1366**	**1791**	947	**2982**	0				
Slovakia	56	**971**	776	**1969**	292	894	**1351**	**1430**	**1094**	554	**1251**	162	**1739**	785	921	**1086**	821	**1378**	**988**	534	**2355**	805	0			
Slovenia	279	921	794	**1992**	449	**1080**	**1638**	**1714**	**969**	724	**1176**	382	**1696**	490	1225	**1380**	742	**1127**	**991**	835	**2102**	926	306	0		
Spain	**1813**	**1317**	**2259**	**3291**	**1775**	**2075**	**2898**	**2953**	**1052**	**1870**	**2375**	**1979**	**1450**	**1367**	**2666**	**2717**	**1281**	**1670**	**1482**	**2294**	504	**2479**	**1863**	**1602**	0	
Sweden	**1242**	**1283**	**1885**	**2908**	**1055**	523	380	397	**1548**	813	**2409**	**1319**	**1634**	**1978**	678	443	**1325**	**2618**	**1128**	809	**2994**	**1744**	**1246**	**1497**	**2596**	0
UK	***1237***	***321***	***2018***	***3221***	***1035***	***958***	***1788***	***1826***	***343***	***932***	***2394***	***1454***	***466***	***1434***	***1728***	***1681***	***490***	***2088***	***358***	***1452***	***1586***	***2095***	***1291***	***1231***	***1263***	***1437***
