# Supplementary material for: Livestock trade networks for guiding animal health surveillance
Source: BMC Vet Res. 2015 Apr 1;11:82. doi: 10.1186/s12917-015-0354-4 (PMC4411738; doi:10.1186/s12917-015-0354-4)
Supplement: Additional file 4: — The proportions of national animal imports and exports compared with the national population. These data are displayed in separate tables for each species. [file 12917_2015_354_MOESM4_ESM.pdf]

Additional file 4.

Table 1. Proportions of cattle imports and exports compared with the cattle population of the country importing or exporting the animal. The numbers in bold indicate countries where ≥10% of the national population is imported.

Direction of trade	Purpose of trade	Countries																									
		Austria	Belgium	Bulgaria	Cyprus	Czech Republic	Denmark	Estonia	Finland	France	Germany	Greece	Hungary	Ireland	Italy	Lithuania	Latvia	Luxembourg	Malta	Netherlands	Poland	Portugal	Romania	Slovakia	Slovenia	Spain	Sweden	UK
Import	All cattle breeding	0.04	2.04	0.32	0.00	0.12	0.01	0.37	0.01	0.02	0.22	2.30	1.01	0.08	2.30	0.17	0.51	0.93	2.13	3.13	0.25	0.22	0.40	0.61	1.29	0.92	0.00	0.39
Export	All cattle breeding	1.45	0.58	0.00	0.00	2.17	0.79	0.04	0.02	0.40	0.36	0.07	0.38	1.12	0.13	0.02	0.07	3.33	0.00	1.42	2.42	0.09	0.06	4.33	0.01	0.05	0.02	0.07
Import	All cattle fattening	0.03	6.42	0.37	0.00	0.16	0.00	0.00	0.00	0.35	0.38	5.98	3.99	0.11	**18.66**	0.31	0.03	1.06	1.11	**21.34**	0.20	0.03	0.14	3.12	0.91	9.14	0.00	0.14
Export	All cattle fattening	2.87	9.36	0.27	0.00	4.56	1.34	**12.80**	0.00	6.82	4.30	0.02	0.82	1.94	0.52	8.23	**11.03**	7.43	0.00	0.36	2.80	1.66	6.53	0.55	3.02	0.97	0.00	0.33
Import	All cattle Slaughter	5.10	3.43	0.04	0.00	0.10	0.00	0.00	0.00	0.30	0.70	0.16	1.26	0.16	1.13	2.91	1.61	0.43	0.00	2.20	0.20	0.23	0.00	0.65	0.16	0.46	0.00	0.30
Export	All cattle Slaughter	0.20	3.26	0.01	0.00	5.12	0.06	3.03	0.00	0.30	0.29	0.12	1.29	0.49	0.00	0.10	1.83	**13.13**	0.00	3.25	0.33	1.75	0.29	7.90	7.10	0.48	0.00	0.10
Import	All cattle Other	0.00	0.51	0.00	0.00	0.00	0.00	0.00	0.00	0.01	0.05	0.01	0.03	0.00	0.27	0.00	0.03	0.03	0.00	0.24	0.01	0.00	0.00	0.01	0.00	0.18	0.00	0.00
Export	All cattle Other	1.14	0.01	0.01	0.00	0.01	0.00	0.00	0.00	0.00	0.16	0.00	0.01	0.00	0.01	1.68	0.00	0.00	0.00	0.01	0.00	0.02	0.00	0.00	0.00	0.02	0.00	0.00

Table 2. Proportions of pig imports and exports compared with the pig population of the country importing or exporting the animal. The numbers in bold indicate countries where ≥10% of the national population is imported.

Direction of trade	Purpose of trade	Countries																									
		Austria	Belgium	Bulgaria	Cyprus	Czech Republic	Denmark	Estonia	Finland	France	Germany	Greece	Hungary	Ireland	Italy	Lithuania	Latvia	Luxembourg	Malta	Netherlands	Poland	Portugal	Romania	Slovakia	Slovenia	Spain	Sweden	UK
Import	Breeding	0.25	**13.17**	1.48	0.08	5.17	0.00	0.01	0.01	0.20	0.05	**324.41**	0.09	6.05	0.01	**52.68**	0.01	4.65	3.55	0.00	0.31	0.00	**14.48**	1.02	**70.98**	0.03	2.75	3.42
Export	Breeding	0.00	0.00	0.00	0.00	0.00	0.00	0.00	0.00	0.00	0.00	0.00	0.00	0.22	0.00	0.00	0.00	0.00	0.00	0.00	0.00	0.00	0.00	0.00	0.00	0.00	0.01	0.00
Import	Fattening	4.55	0.73	1.30	0.00	**30.39**	0.00	0.00	0.00	0.41	**22.58**	0.29	9.40	0.28	6.11	0.85	0.00	**62.12**	0.00	0.42	**12.94**	1.63	7.78	**13.92**	**12.59**	0.14	0.00	2.70
Export	Fattening	1.50	2.01	0.00	0.00	1.12	**65.84**	0.00	0.00	0.39	4.17	0.01	2.78	7.53	0.00	9.05	**14.32**	8.41	2.59	2.57	0.00	0.25	0.00	0.01	**14.55**	0.24	0.00	0.10
Import	Slaughter	**17.72**	9.62	0.13	0.00	**10.06**	0.00	0.05	0.00	1.48	**18.37**	0.08	**24.45**	0.40	3.16	3.08	5.71	**47.64**	0.00	4.90	5.35	**76.43**	3.46	0.13	**76.55**	1.19	0.00	**13.71**
Export	Slaughter	0.26	**14.88**	0.00	0.00	**15.49**	3.08	0.00	0.00	4.24	4.85	0.05	9.94	**38.20**	0.12	8.38	0.20	**88.77**	0.00	**41.50**	0.53	6.96	0.00	6.52	**81.06**	6.70	1.07	0.15
Import	Other	0.02	0.00	0.00	0.00	0.08	0.00	0.00	0.00	0.01	0.01	0.00	0.01	0.00	0.00	0.00	0.00	0.12	0.00	0.00	0.00	0.03	0.00	0.12	0.00	0.00	0.00	0.00
Export	Other	0.00	0.00	0.00	0.00	0.00	0.02	0.00	0.00	0.00	0.01	0.00	0.01	0.00	0.00	0.00	0.00	0.00	0.00	0.01	0.00	0.00	0.00	0.00	0.00	0.00	0.00	0.00

Table 3. Proportions of sheep imports and exports compared with the sheep population of the country importing or exporting the animal, NAs occurred when there was a shortage of data concerning the national population of sheep. The numbers in bold indicate countries where ≥10% of the national population is imported.

Direction of trade	Purpose of trade	Countries																									
		Austria	Belgium	Bulgaria	Cyprus	Czech Republic	Denmark	Estonia	Finland	France	Germany	Greece	Hungary	Ireland	Italy	Lithuania	Latvia	Luxembourg	Malta	Netherlands	Poland	Portugal	Romania	Slovakia	Slovenia	Spain	Sweden	UK
Import	Breeding	0.84	NA	0.79	0.12	NA	NA	NA	NA	0.02	0.07	0.13	0.01	0.11	0.08	0.42	NA	NA	0.04	0.12	0.22	0.25	0.03	0.00	NA	0.05	0.00	0.00
Export	Breeding	0.76	NA	0.00	0.00	NA	NA	NA	NA	0.13	0.11	0.00	0.09	0.01	0.00	0.00	NA	NA	0.00	0.20	0.34	0.02	0.08	0.00	NA	0.14	0.02	0.02
Import	Fattening	1.57	NA	**31.71**	0.00	NA	NA	NA	NA	1.58	0.94	3.01	6.04	0.05	5.02	1.60	NA	NA	0.00	4.28	0.00	0.24	0.02	0.00	NA	0.46	0.00	0.00
Export	Fattening	5.73	NA	0.22	0.00	NA	NA	NA	NA	9.13	4.55	0.08	**45.78**	0.51	0.04	0.00	NA	NA	0.00	**20.72**	**31.50**	4.50	**16.35**	0.28	NA	2.33	0.02	2.41
Import	Slaughter	1.66	NA	**21.60**	0.00	NA	NA	NA	NA	2.28	2.67	0.35	1.74	**13.60**	**10.85**	0.48	NA	NA	0.00	1.85	2.42	0.88	0.00	0.00	NA	2.80	0.02	0.00
Export	Slaughter	2.10	NA	0.18	0.00	NA	NA	NA	NA	8.79	1.23	0.02	16.24	0.01	0.01	0.00	NA	NA	0.00	**19.26**	**21.84**	1.45	6.37	0.28	NA	1.56	0.00	2.13
Import	Other	0.33	NA	0.05	0.00	NA	NA	NA	NA	0.03	0.02	0.00	0.00	0.00	0.41	0.02	NA	NA	0.00	0.05	0.00	0.03	0.01	0.00	NA	0.01	0.00	0.00
Export	Other	0.13	NA	0.04	0.00	NA	NA	NA	NA	0.00	0.04	0.00	2.85	0.01	0.03	0.00	NA	NA	0.00	0.02	0.00	0.06	0.00	0.00	NA	0.01	0.00	0.00

Table 4. Proportions of goat imports and exports compared with the goat population of the country importing or exporting the animal, NAs occurred when there was a shortage of data concerning the national population of goats. The numbers in bold indicate countries where ≥10% of the national population is imported.

Direction of trade	Purpose of trade	Countries																									
		Austria	Belgium	Bulgaria	Cyprus	Czech Republic	Denmark	Estonia	Finland	France	Germany	Greece	Hungary	Ireland	Italy	Lithuania	Latvia	Luxembourg	Malta	Netherlands	Poland	Portugal	Romania	Slovakia	Slovenia	Spain	Sweden	UK
Import	Breeding	0.17	NA	0.05	0.00	NA	NA	NA	NA	0.01	0.44	0.05	0.03	NA	0.14	0.01	NA	NA	0.06	0.01	0.07	0.73	0.17	0.15	NA	0.04	NA	NA
Export	Breeding	1.27	NA	0.00	0.00	NA	NA	NA	NA	0.13	0.35	0.00	0.37	NA	0.01	0.01	NA	NA	0.00	0.10	0.00	0.07	0.09	0.02	NA	0.22	NA	NA
Import	Fattening	0.03	NA	0.00	0.00	NA	NA	NA	NA	0.33	0.01	0.01	0.00	NA	0.26	0.00	NA	NA	0.00	0.14	0.00	0.23	0.00	0.00	NA	0.00	NA	NA
Export	Fattening	0.88	NA	0.00	0.00	NA	NA	NA	NA	0.01	2.30	0.00	1.60	NA	0.00	0.00	NA	NA	0.00	0.16	0.00	0.00	0.04	0.00	NA	0.05	NA	NA
Import	Slaughter	0.06	NA	0.00	0.00	NA	NA	NA	NA	0.28	0.15	0.00	0.01	NA	1.28	0.00	NA	NA	0.00	0.09	0.00	3.72	0.00	0.00	NA	0.92	NA	NA
Export	Slaughter	2.89	NA	0.00	0.00	NA	NA	NA	NA	1.68	0.05	0.00	6.85	NA	0.00	0.00	NA	NA	0.00	1.93	0.00	0.00	0.01	0.84	NA	0.56	NA	NA
Import	Other	0.06	NA	0.00	0.00	NA	NA	NA	NA	0.02	0.00	0.00	0.00	NA	0.01	0.05	NA	NA	0.00	0.00	0.01	0.09	0.00	0.00	NA	0.00	NA	NA
Export	Other	0.16	NA	0.00	0.00	NA	NA	NA	NA	0.00	0.03	0.00	0.07	NA	0.02	0.00	NA	NA	0.00	0.00	0.00	0.00	0.00	0.00	NA	0.01	NA	NA

Table 5. Proportions of poultry imports and exports compared with the poultry population of the country importing or exporting the animal, NAs occurred when there was a shortage of data concerning the national population of poultry. The numbers in bold indicate countries where ≥10% of the national population is imported.

Direction of trade	Purpose of trade	Countries																										
		Austria	Belgium	Bulgaria	Cyprus	Czech Republic	Denmark	Estonia	Finland	France	Germany	Greece	Hungary	Ireland	Italy	Lithuania	Latvia	Luxembourg	Malta	Netherlands	Poland	Portugal	Romania	Slovakia	Slovenia	Spain	Sweden	UK
Import	Breeding	1.00	4.80	2.54	1.94	**11.05**	1.18	NA	0.15	0.49	1.33	NA	3.90	3.45	5.79	**11.89**	0.28	NA	NA	4.27	3.81	5.85	18.39	1.90	NA	0.98	NA	0.39
Export	Breeding	0.42	8.34	0.00	0.00	**18.10**	4.59	NA	1.08	2.39	2.53	NA	2.95	0.49	6.93	0.00	0.00	NA	NA	2.25	0.08	0.16	0.72	0.02	NA	1.99	NA	0.78
Import	Slaughter	9.52	3.72	0.54	0.00	0.96	0.00	NA	0.00	0.09	3.60	NA	2.12	7.55	0.38	**11.29**	0.03	NA	NA	2.23	2.92	0.05	0.03	0.90	NA	0.35	NA	0.25
Export	Slaughter	0.87	2.83	0.00	0.00	**10.06**	9.01	NA	0.00	0.45	2.54	NA	0.76	3.25	0.02	5.55	0.20	NA	NA	1.33	0.25	0.79	1.57	3.91	NA	0.04	NA	0.58
Import	Other	4.13	4.51	1.83	0.23	9.04	0.99	NA	0.12	0.10	1.94	NA	3.17	**20.62**	0.80	**21.93**	0.29	NA	NA	1.46	2.73	1.32	**16.15**	**10.06**	NA	0.67	NA	1.06
Export	Other	23.28	1.84	**28.79**	0.00	2.04	13.26	NA	0.71	0.87	4.34	NA	7.05	9.07	0.04	5.17	0.00	NA	NA	3.03	0.72	0.01	0.46	0.05	NA	0.08	NA	1.38
